# Public preparedness and knowledge about emergency medicine: A study across 6 countries

**DOI:** 10.1097/MD.0000000000043217

**Published:** 2025-07-11

**Authors:** Esra’ O. Taybeh, Abdallah Y. Naser, Adnan Taybeh, Zahra K. Alsairafi, Hassan Alwafi, Sami Qadus, Rania Itani, Alaa A. Alsharif, Ahmed M. Al Rajeh, Jaber S. Alqahtani, Abdulelah M. Aldhahir, Anan S. Jarab, Saeed Alqahtani, Abdolelah Jaradat, Louai Saloumi, Yosra J. Alhartani, Asaleh El-Qasem, Amer Hamad Issa Abukhalaf, Salman Alzayani, Roua Awni Attallah Aldala’een, Ahmad H. Aburizeq, Ahmad Khaleel Hijazi, Jamal Alyoussef Alkrad, Mohamed Bahlol

**Affiliations:** a Department of Applied Pharmaceutical Sciences and Clinical Pharmacy, Faculty of Pharmacy, Isra University, Amman, Jordan; b Al Bashir Hospital, Ministry of Health, Amman, Jordan; c Department of Pharmacy Practice, Faculty of Pharmacy, Kuwait University, Hawalli, Kuwait; d Department of Pharmacology and Toxicology, College of Medicine, Umm Al-qura University, Makkah, Saudi Arabia; e Department of Pharmacy, Faculty of Health Sciences, American University of Madaba, Madaba, Jordan; f Pharmacy Practice Department, Faculty of Pharmacy, Beirut Arab University, Riad El Solh, Beirut, Lebanon; g Department of Pharmacy Practice, Faculty of Pharmacy, Princess Nourah Bint Abdulrahman University, Riyadh, Saudi Arabia; h Department of Respiratory Care, College of Applied Medical Sciences, King Faisal University, Al-Ahsa, Saudi Arabia; i Department of Respiratory Care, Prince Sultan Military College of Health Sciences, Dammam, Saudi Arabia; j Department of Nursing, Respiratory Therapy Program, College of Nursing and Health Sciences, Jazan University, Jazan, Saudi Arabia; k Health Research Center, Jazan University, Jazan, Saudi Arabia; l Department of Clinical Pharmacy, Faculty of Pharmacy, Jordan University of Science and Technology, Irbid, Jordan; m Department of Emergency Medical Services, Prince Sultan Military College of Health Sciences, Dammam, Saudi Arabia; n Faculty of Pharmacy, University of Jordan, Amman, Jordan; o Nieri Department of Construction and Real Estate Development, Development and Planning, Clemson University, Clemson, SC; p Department of Family and Community Medicine, Arabian Gulf University, Manama, Bahrain; q Department of Pharmacy Practice and Clinical Pharmacy, Specialty of Pharmaceutical Management and Economics, Egyptian Russian University, Cairo, Egypt.

**Keywords:** Bahrain, Egypt, emergency health, Jordan, knowledge, Kuwait, Lebanon, preparedness, Saudi Arabia

## Abstract

Low health literacy is associated with poor health outcomes, inefficient use of healthcare resources, higher mortality risk, and increased costs. The aim of this research is to explore public awareness and preparedness regarding various treatment options for acute medical events and to assess the competence in assessing the urgency of such medical situations in 6 Middle Eastern countries namely, Jordan, Saudi Arabia, Egypt, Lebanon, Kuwait, and Bahrain. This research involved a cross-sectional survey study design to assess public knowledge and preparedness about emergency medicine using an online questionnaire distributed through accessible online channels between March 1, 2024 until September 11, 2024. A total of 4909 participants were involved in this research. The questionnaire was developed based on a thorough review of existing literature related to public knowledge and public preparedness of emergency medicine. Binary logistic regression analysis was used to identify significant predictors of insufficient and problematic emergency health literacy (EHL) levels. Overall, the most commonly known emergency care service was identifying emergency practice nearby (81.4%). The least commonly known emergency care service was identifying the rescue service (48.1%). The highest degree of difficulty reported by the study participants was the difficulties they face to evaluate when to use emergency medical services (39.6%). The mean public EHL score for the study sample was 2.77 (standard deviation [SD]: 0.44) out of 3. The mean public EHL score ranged between 2.69 (SD: 0.50) for Kuwait and 2.89 (SD: 0.56) for Bahrain. The majority of the study participants (75.1%) demonstrated problematic EHL. Males, young participants aged 24 to 30 years and elderly participants aged 61 years and older, highly educated individuals, high income level individuals, and those who work in the healthcare sector were less likely to have inadequate and problematic EHL levels (*P* < .05). The findings of the present study indicate limited emergency public EHL among 6 of the Middle Eastern countries, highlighting the need for interventions to promote public knowledge and capabilities. Educational campaigns that promote EHL should target females, those with low education and socioeconomic status, those in age range of 30 to 60 years, and those who do not work in health sector.

## 1. Introduction

Health emergencies are defined as unforeseen events that seriously affect health, causing significant damage to the health and well-being. These include major infectious disease outbreaks, major food and occupational poisoning events, unexplained diseases, and incidents involving biological, chemical, and natural disasters such as floods, earthquakes, and fires.^[1]^ Within the context of these emergencies, emergency care is crucial as it is provided within the first few hours following the onset of an acute medical condition that poses a life-threatening risk.^[2]^

Healthcare utilization, particularly emergency department (ED) visits, is increasing rapidly.^[3–7]^ This place an added burden on healthcare systems through excessive healthcare spending and unnecessary testing and treatment.^[8]^ This strain is exacerbated when a large portion of these ED visits are preventable.^[9]^ Approximately 37% and 50% of total ED visits in the United States and in Australia are for non-urgent conditions, respectively.^[10]^ Moreover, reports of “unnecessary ambulance use” have been received from England, Canada, and South Africa.^[11]^ Factors such as race, age, educational level, healthcare background, and prior first-aid training are associated with the misuse of emergency services.^[11]^ Importantly, low levels of public health literacy have been linked with more frequent utilization of health services and emergency medicine.^[12]^

Health literacy refers to the extent to which individuals can obtain, process, and comprehend essential health information and services required for health decisions.^[13]^ Over the past recent decades, increasing attention to the concept of health literacy has been given due to its significant benefits for both individual and public health as well as for the sustainability of healthcare systems through educating individuals to take greater responsibility for managing their own health and making more effective use of health services.^[14]^

Population-based studies from European countries have shown that between one-third and two-third thirds of the population report inadequate health literacy.^[15]^ Low emergency health literacy (EHL) is associated with challenges in understanding health information, limited knowledge of the disease, and lower medication adherence, all of which contribute to poor health outcomes, inefficient use of healthcare resources, higher mortality risk, and increased costs.^[14]^ Evidence suggests that improving health literacy can overcome such challenges therefore, many countries have prioritized health literacy in their policies and practices. In Norway, for example, 89% of middle-school students receive basic life support training.^[16]^ Similarly, around 70% of the Republic of Slovenia population participates in cardiopulmonary resuscitation (CPR) courses.^[17]^ In Switzerland, every resident undergoes emergency training and in Australia, the rate of emergency training reaches approximately 60%.^[18,19]^ A previous study in Jordan reported that schoolteachers in Jordan have a limited understanding of CPR. Nevertheless, the research participants exhibited a favorable perspective regarding the execution of CPR.^[20]^ Moreover, a recent systematic review and meta-analysis study examined CPR knowledge among 30,308 non-healthcare providers in different Arab countries namely Syria, Saudi Arabia, Iraq, Jordan, Egypt, Lebanon, Kuwait, and Oman and found that 55.0% of the participants demonstrated previous knowledge of CPR. Besides, the level of CPR knowledge varied significantly between students, general population, and teachers.^[21]^

Recent research from multiple the United Kingdom (UK) and Australia showed rising trends of hospitalization across different disease areas including respiratory diseases, heart diseases, diabetes mellitus, and infectious diseases.^[22–26]^ There are only a few studies specifically addressing public EHL and preparedness about emergency care and emergency medicine in countries with a developing emergency care system. In this regard, the aim of this research is to explore public awareness and preparedness regarding various treatment options for acute medical events and to assess the competence in assessing the urgency of such medical situations in 6 Middle Eastern countries namely, Jordan, Saudi Arabia, Egypt, Lebanon, Kuwait, and Bahrain.

## 2. Methods

### 2.1. Study design

This research involved a cross-sectional survey study design to assess public knowledge and preparedness about emergency medicine across 6 Middle Eastern countries namely, Jordan, Saudi Arabia, Egypt, Lebanon, Kuwait, and Bahrain using an online questionnaire distributed through accessible social media channels between March 1, 2024 until September 11, 2024.

### 2.2. Sampling technique

A convenience sampling technique was utilized to recruit the targeted study population. The general public formed the study population for this research. The questionnaire was distributed online using the multiple social media platforms (namely, WhatsApp, Facebook, and X). This platform was chosen for its ease of use and its ability of tracking and analysis of responses by each country. Besides, social media platforms were utilized to reach participants from different socioeconomic backgrounds across different countries. The inclusion criteria were adult individuals aged 18 years and above, currently living in one of the participating countries. In this study, we did not restrict the demographic characteristics for our targeted study population to specific gender, or specific demographic groups in order to enhance the generalizability of our study findings. The countries were selected within the middle east region based on their geographic diversity and varying healthcare systems to provide a comprehensive overview of public knowledge.

### 2.3. Questionnaire development

The questionnaire was developed based on a thorough review of existing literature related to public knowledge and public preparedness of emergency medicine.^[12,15]^ The literature review directed the development of questions covering key areas including urgency evaluation and decision-making, knowledge of emergency services, and knowledge of emergency medicines. Therefore, the questionnaire was divided into 4 sections. Section one collected sociodemographic data. Section 2 composed of 3 questions with 4-item Likert scale from “very easy” to “very difficult” and measured the difficulty of population of urgency evaluation and decision-making after an acute medical case. Section 3 composed of 3 questions assessed public knowledge of emergency services using yes/no answers. The principal component analysis was conducted for the original questionnaire and revealed that the 3 items that examined public knowledge of emergency services loaded on 1 factor (Eigenvalue 1.84; explained variance 61.37%; loadings 0.72 to 0.82; Cronbach Alpha 0.68). The last section (16 questions) explored public knowledge of emergency medicines.

### 2.4. Questionnaire translation

The questionnaire was firstly designed in English and then translated using forward-backward translation technique to Arabic to be user-friendly and included a combination of Likert scale items, and yes/no questions. Before the survey, participants received a short introduction outlining the research aim and emphasizing voluntary participation. Participants also were informed about confidentiality of data and consented before accessing the questions. This format aimed to capture a range of responses and provide detailed insights into participants’ knowledge. Information on emergency medical services, rescue services, and awareness of the national emergency number was examined in this research. As emergency systems could vary between the participating countries, the questionnaire was accurately translated into Arabic with explanations to ensure respondents grasped the differences. Emergency medical services were referred to as prehospital medical care and ambulance transport, and rescue operations as activities such as extrication from fire rescues, crashes, and search-and-rescue missions, which might be operated by civil defense or fire departments. Although the services overlap in some countries, they differ in others, and the survey was adjusted accordingly.

Emergency medical services systems are not the same across the participating countries in this research. In Jordan, medical services adopt hybrid model that combine both the American and the European Emergency medical services system.^[27]^ While in Saudi Arabia, Kuwait, and Bahrain the emergency medical services follow the American system.^[28–30]^ At the same time, Egypt follows government-centralized model through the Egyptian Ambulance Organization.^[31,32]^ Besides, Lebanon adopts both the European volunteer-based system and the American system.^[33]^

### 2.5. Questionnaire validity and reliability

To ensure the validity of the questionnaire, the initial draft of the questionnaire was reviewed by a panel of experts in public health within academic and health institutions. These experts assessed the relevance and comprehensiveness of the questions to match the research question and their feedback was used to refine the questionnaire items. After that, a pilot version of the questionnaire was administered to a small sample of 10 participants and was included in the main study since no further revisions were made on the questionnaire. The final draft of the questionnaire was subjected to a reliability analysis to assess internal consistency, with Cronbach alpha coefficient of 0.907, which indicates excellent reliability.

### 2.6. Data analysis

Responses were exported to SPSS (version 29) analysis software for analysis. Descriptive statistics were used to summarize participants’ demographics and knowledge and presented as frequencies and percentages. The normality of continuous variables was checked through histogram, which confirmed that EHL score was normally distributed. Therefore, the mean and standard deviation (SD) were used to present continuous variables. The internal reliability of the questionnaire items was examined using Cronbach alpha test. Binary logistic regression analysis was used to identify significant predictors of insufficient and problematic EHL levels. The dummy variable in the binary logistic regression model was defined as a mean EHL score below 2 (a score of 2 was used to define the dummy variable for the dependent variable in the logistic regression analysis model) The findings of the logistic regression analysis were presented as odds ratio with its corresponding 95% confidence interval. The significance level was assigned as *P*-value <.05.

### 2.7. Ethical approval

The ethical approval for the present research was gained from the Ethical Approval Committee at Isra University (SREC/24/03/099). Informed consent was obtained from all participants in the introductory page of the questionnaire, and anonymity was ensured by not collecting personally identifiable information.

## 3. Results

### 3.1. Sociodemographic characteristics of the study participants

A total of 4909 participants were involved in this research. More than half of the study participants (62.9%) were females and aged 18 to 23 years (60.2%). Around 70.0% of the study participants were single. Around 52.6% of the study participants reported that they have bachelor degree. The monthly income category was <700 USD for 50.5% of the study participants. Around 38.3% of the study participants were universities students. Smokers comprised 16.9% of the study participants. Around 12.4% of the study participants reported that they have comorbidities history, Table 1.

**Table 1:** Sociodemographic characteristics of the study participants.

Variable	Overall (n = 4909)	Jordan (n = 1607)	Saudi Arabia (n = 1430)	Egypt (n = 1308)	Lebanon (n = 405)	Kuwait (n = 104)	Bahrain (n = 55)
Gender							
** **Females	3090 (62.9%)	1384 (86.1%)	477 (33.4%)	816 (62.4%)	311 (76.8%)	72 (69.2%)	30 (54.5%)
** **Males	1819 (37.1%)	223 (13.9%)	953 (66.6%)	492 (37.6%)	94 (23.2%)	32 (30.8%)	25 (45.5%)
Age groups							
** **18–23 yr	2954 (60.2%)	1304 (81.1%)	572 (40.0%)	854 (65.3%)	204 (50.4%)	14 (13.5%)	6 (10.9%)
** **24–30 yr	789 (16.1%)	196 (12.2%)	313 (21.9%)	197 (15.1%)	53 (13.1%)	23 (22.1%)	7 (12.7%)
** **31–40 yr	399 (8.1%)	33 (2.1%)	245 (17.1%)	52 (4.0%)	43 (10.6%)	12 (11.5%)	14 (25.5%)
** **41–50 yr	443 (9.0%)	27 (1.7%)	214 (15.0%)	103 (7.9%)	48 (11.9%)	36 (34.6%)	15 (27.3%)
** **51–60 yr	235 (4.8%)	28 (1.7%)	63 (4.4%)	86 (6.6%)	36 (8.9%)	12 (11.5%)	10 (18.2%)
** **61 yr and older	89 (1.8%)	19 (1.2%)	23 (1.6%)	16 (1.2%)	21 (5.2%)	7 (6.7%)	3 (5.5%)
Marital status							
** **Single	3425 (69.8%)	1374 (85.5%)	788 (55.1%)	970 (74.2%)	246 (60.7%)	35 (33.7%)	12 (21.8%)
** **Married	1367 (27.8%)	188 (11.7%)	607 (42.4%)	322 (24.6%)	149 (36.8%)	60 (57.7%)	41 (74.5%)
** **Divorced	63 (1.3%)	18 (1.1%)	29 (2.0%)	5 (0.4%)	4 (1.0%)	6 (5.8%)	1 (1.8%)
** **Widowed	54 (1.1%)	27 (1.7%)	6 (0.4%)	11 (0.8%)	6 (1.5%)	3 (2.9%)	1 (1.8%)
Education level							
** **Secondary school or lower	1420 (28.9%)	774 (48.2%)	390 (27.3%)	103 (7.9%)	9 (16.4%)	24 (23.1%)	9 (16.4%)
** **Bachelor degree	2581 (52.6%)	762 (47.4%)	896 (62.7%)	647 (49.5%)	22 (40.0%)	58 (55.8%)	22 (40.0%)
** **Higher education	908 (18.5%)	71 (4.4%)	144 (10.1%)	558 (42.7%)	24 (43.6%)	22 (21.2%)	24 (43.6%)
Monthly income category							
** **<700 USD	2478 (50.5%)	972 (60.5%)	255 (17.8%)	930 (71.1%)	315 (77.8%)	3 (2.9%)	3 (5.5%)
** **700–1500 USD	989 (20.1%)	410 (25.5%)	240 (16.8%)	264 (20.2%)	61 (15.1%)	10 (9.6%)	4 (7.3%)
** **1500–2000 USD	460 (9.4%)	107 (6.7%)	254 (17.8%)	70 (5.4%)	18 (4.4%)	7 (6.7%)	4 (7.3%)
** **More than 2000 USD	982 (20.0%)	118 (7.3%)	681 (47.6%)	44 (3.4%)	11 (2.7%)	84 (80.8%)	44 (80.8%)
Employment status							
** **Retired	149 (3.0%)	14 (0.9%)	64 (4.5%)	30 (2.3%)	14 (3.5%)	14 (13.5%)	13 (23.6%)
** **Unemployed	1347 (27.4%)	766 (47.7%)	247 (17.3%)	203 (15.5%)	119 (29.4%)	8 (7.7%)	4 (7.3%)
** **Working in healthcare sector	532 (10.8%)	75 (4.7%)	227 (15.9%)	159 (12.2%)	39 (9.6%)	15 (14.4%)	17 (30.9%)
** **Student	1882 (38.3%)	600 (37.3%)	481 (33.6%)	663 (50.7%)	119 (29.4%)	15 (14.4%)	4 (7.3%)
** **Working outside healthcare sector	999 (20.4%)	152 (9.5%)	411 (28.7%)	253 (19.3%)	114 (28.1%)	52 (50.0%)	17 (30.9%)
Current smoker (Yes)	829 (16.9%)	325 (20.2%)	244 (17.1%)	119 (9.1%)	107 (26.4%)	24 (23.1%)	10 (18.2%)
Have comorbidities history (Yes)	608 (12.4%)	156 (9.7%)	180 (12.6%)	146 (11.2%)	74 (18.3%)	30 (28.8%)	22 (40.0%)

### 3.2. Public knowledge about emergency care services

**Figure 1.**
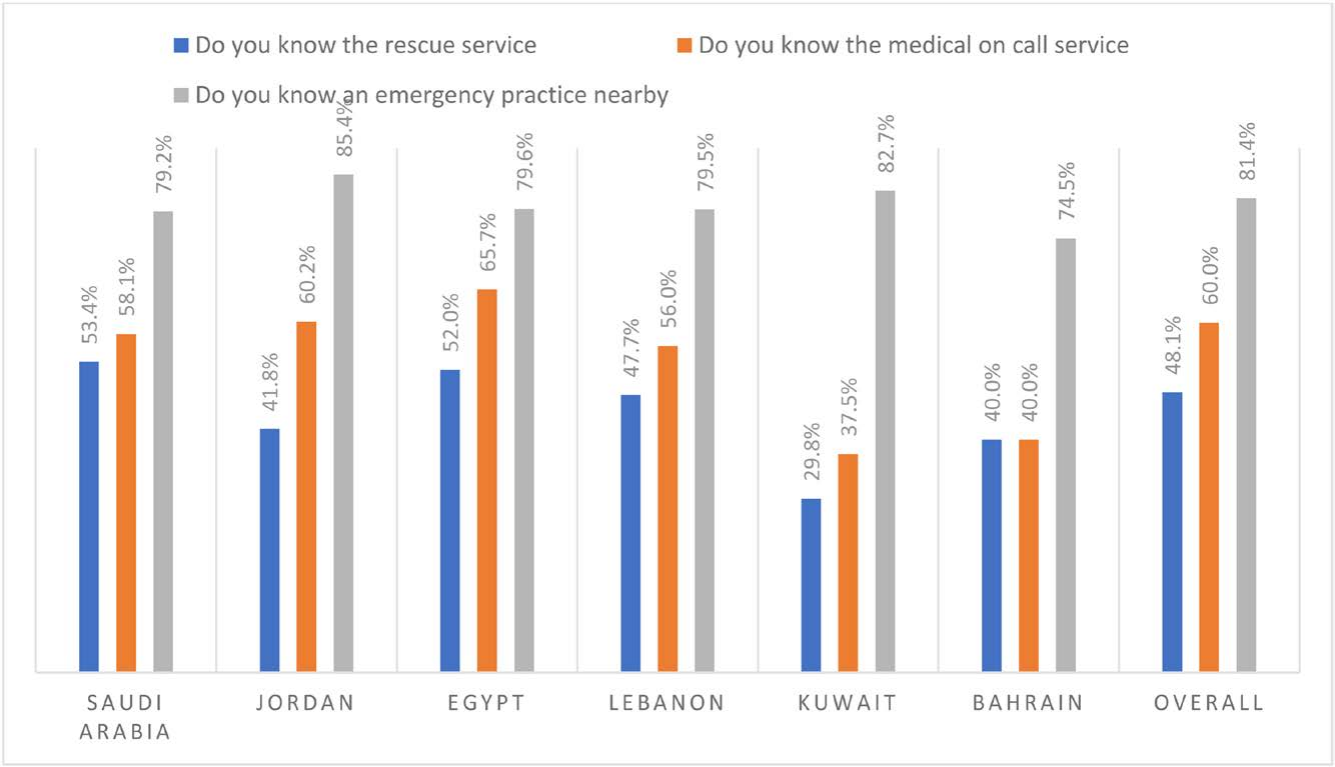
Public knowledge about emergency care services.

Overall, the most commonly known emergency care service was identifying emergency practice nearby (81.4%). The least commonly known emergency care service was identifying the rescue service (48.1%). Figure 1 below presents public knowledge about emergency care services stratified by country.

### 3.3. Capabilities of dealing with emergency cases

**Figure 2.**
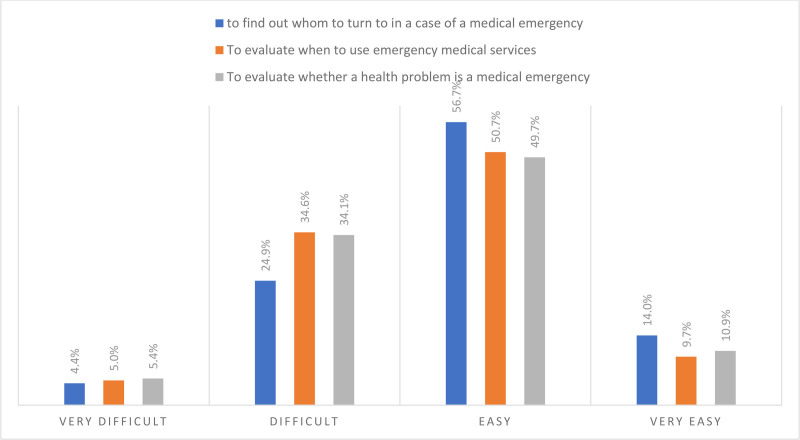
Overall capabilities of dealing with emergency cases.

The highest degree of difficulty reported by the study participants was the difficulties they face to evaluate when to use emergency medical services (39.6%). The lowest degree of difficulty reported by the study participants was concerning the difficulties they face find out whom to turn to in a case of a medical emergency (29.3%). Figure 2 below presents the overall capabilities of dealing with emergency cases.

**Figure 3.**
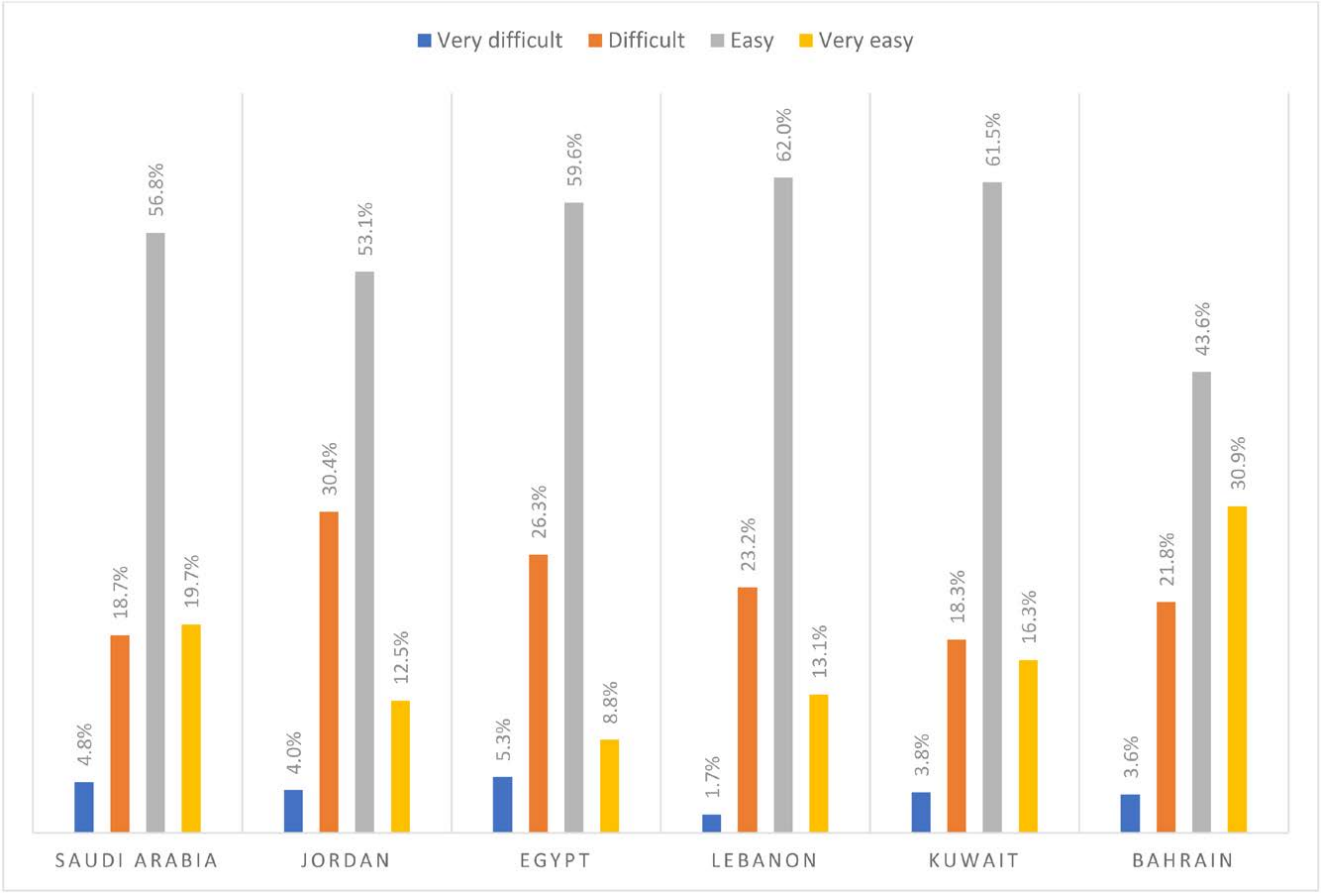
Difficulties to find out whom to turn to in a case of a medical emergency stratified by country.

The perceived difficulty degree concerning finding out whom to turn to in a case of a medical emergency ranged between 22.1% (for Kuwait) and 34.4% (for Jordan). In Kuwait, around 3.8% and 18.3% of the respondents reported that it is very difficult to difficult for them to finding out whom to turn to in a case of a medical emergency, respectively. In Jordan, around 4.0% and 30.4% of the respondents reported that it is very difficult to difficult for them to finding out whom to turn to in a case of a medical emergency. Figure 3 below presents the perceived difficulties to find out whom to turn to in a case of a medical emergency stratified by country.

**Figure 4.**
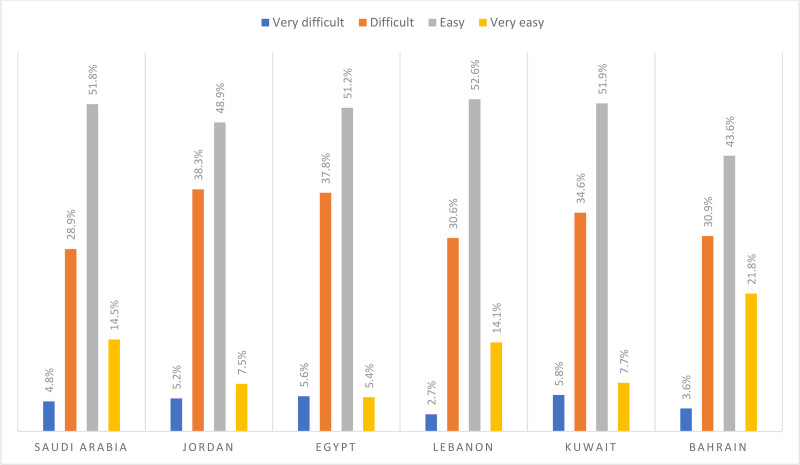
Difficulties to evaluate when to use emergency medical services stratified by country.

The perceived difficulty degree concerning evaluating when to use emergency medical services ranged between 33.3% (for Lebanon) and 43.6% (for Jordan). In Lebanon, around 2.7% and 30.6% of the respondents reported that it is very difficult to difficult for them to evaluate when to use emergency medical services, respectively. In Jordan, around 5.2% and 38.3% of the respondents reported that it is very difficult to difficult for them to evaluate when to use emergency medical services. Figure 4 below presents difficulties to evaluate when to use emergency medical services stratified by country.

**Figure 5.**
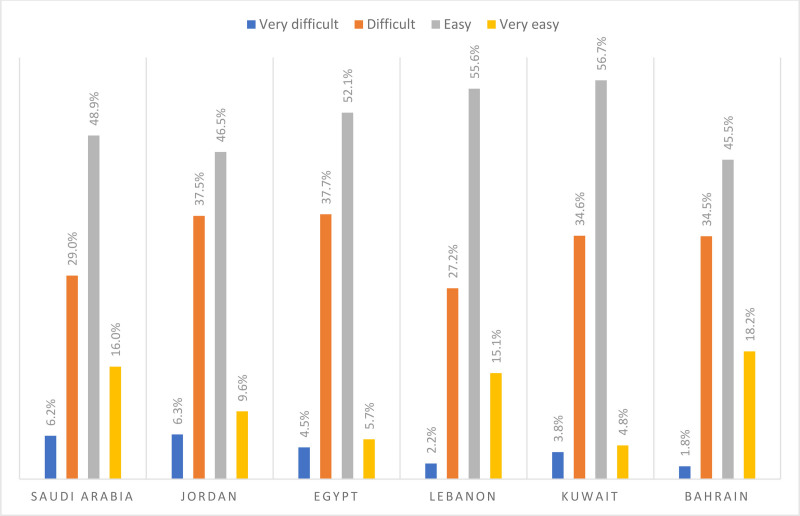
Difficulties to evaluate whether a health problem is a medical emergency stratified by country.

The perceived difficulty degree concerning evaluating whether a health problem is a medical emergency ranged between 29.4% (for Lebanon) and 43.8% (for Jordan). In Lebanon, around 2.2% and 27.2% of the respondents reported that it is very difficult to difficult for them to evaluate whether a health problem is a medical emergency, respectively. In Jordan, around 6.3% and 37.5% of the respondents reported that it is very difficult to difficult for them to evaluate whether a health problem is a medical emergency. Figure 5 below presents difficulties to evaluate whether a health problem is a medical emergency stratified by country.

### 3.4. Public emergency health literacy profile

The mean public EHL score for the study sample was 2.77 (SD: 0.44) out of 3. The mean public EHL score ranged between 2.69 (SD: 0.50) for Kuwait and 2.89 (SD: 0.56) for Bahrain. The majority of the study participants (75.1%) demonstrated problematic EHL. Besides, 4.0% of the study participants showed inadequate EHL level. Only around one-fifth the study participants (21.0%) demonstrated sufficient EHL level (Table 2).

**Table 2:** Public health literacy profile levels and mean score stratified by country.

Health literacy level	Mean score range	Overall	Jordan	Saudi Arabia	Egypt	Lebanon	Kuwait	Bahrain
		Frequency (%)	Frequency (%)	Frequency (%)	Frequency (%)	Frequency (%)	Frequency (%)	Frequency (%)
Inadequate health literacy	0.0–1.0	194 (4.0%)	72 (4.5%)	59 (4.1%)	47 (3.6%)	7 (1.7%)	8 (7.7%)	1 (1.8%)
Problematic health literacy	>1.0–2.0	3686 (75.1%)	1247 (77.6%)	1007 (70.4%)	1056 (80.7%)	264 (65.2%)	75 (72.1%)	37 (67.3%)
Sufficient health literacy	>2.0–3.0	1029 (21.0%)	288 (17.9%)	1430 (25.5%)	205 (15.7%)	134 (33.1%)	21 (20.2%)	17 (30.9%)
** **Mean public health literacy score		2.77 (SD: 0.44)	2.73 (SD: 0.41)	2.83 (SD: 0.50)	2.73 (SD: 0.37)	2.88 (SD: 0.37)	2.69 (SD: 0.50)	2.89 (SD: 0.56)

SD = standard deviation.

Around 48.1% of the study participants confirmed that they are knowledgeable about emergency care services in terms of the rescue service. A higher percentage of the study participants (60.0%) confirmed that they are knowledgeable about emergency care services in terms of the medical on call service. Moreover, the majority of the study participants (81.4%) confirmed that they are knowledgeable about emergency care services in terms of an emergency practice nearby. It worth mentioning that there was a statistically significant difference between the percentage of respondents who confirmed their knowledge of the 3 different aspects that examined emergency health literacy across participants from different countries (*P* < .001). Further details concerning the response to each emergency health literacy question are available in Table 3

**Table 3:** Participants answer to knowledge items stratified by country.

	Knowledge about emergency care services in terms of the rescue service (Percentage of respondents answered yes)	*P*-value	Knowledge about emergency care services in terms of the medical on call service (Percentage of respondents answered yes)	*P*-value	Knowledge about emergency care services in terms of an emergency practice nearby (Percentage of respondents answered yes)	*P*-value
Saudi Arabia	763 (53.4%)	<.001	831 (58.1%)	<.001	1132 (79.2%)	<.001
Jordan	672 (41.8%)		968 (60.2%)		1373 (85.4%)	
Egypt	680 (52.0%)		860 (65.7%)		1041 (79.6%)	
Lebanon	193 (47.7%)		227 (56.0%)		322 (79.5%)	
Kuwait	31 (29.8%)		39 (37.5%)		86 (82.7%)	
Bahrain	22 (40.0%)		22 (40.0%)		41 (74.5%)	
All countries	2361 (48.1%)		2947 (60.0%)		3995 (81.4%)	

### 3.5. Predictors of inadequate and problematic emergency health literacy levels

Table 4 below identifies predictors of inadequate and problematic EHL levels. Males, young participants aged 24 to 30 years and elderly participants aged 61 years and older, highly educated individuals, high income level individuals, and those who work in the healthcare sector were less likely to have inadequate and problematic EHL levels (*P* < .05).

**Table 4:** Predictors of inadequate and problematic health literacy levels.

Variable	Odds ratio (95% confidence interval)	*P*-value
Gender		
** **Females (Reference category)	1.00	
** **Males	0.74 (0.64–0.85)	<.001**
Age groups		
** **18–23 yr (Reference category)	1.00	
** **24–30 yr	0.59 (0.50–0.71)	<.001**
** **31–40 yr	0.87 (0.67–1.12)	.279
** **41–50 yr	1.25 (0.95–1.64)	.105
** **51–60 yr	0.84 (0.61–1.16)	.286
** **61 yr and older	0.61 (0.38–0.98)	.040*
Marital status		
** **Single (Reference category)	1.00	
** **Married	1.03 (0.88–1.20)	.721
** **Divorced	1.42 (0.72–2.81)	.310
** **Widowed	1.05 (0.54–2.05)	.887
Education level		
** **Secondary school or lower (Reference category)	1.00	
** **Bachelor degree	0.74 (0.63–0.87)	<.001**
** **Higher education	0.70 (0.57–0.87)	<.001**
Monthly income category		
** **<700 USD (Reference category)	1.00	
** **700–1500 USD	0.91 (0.76–1.10)	.326
** **1500–2000 USD	0.67 (0.53–0.84)	<.001
** **More than 2000 USD	0.57 (0.48–0.67)	<.001
Employment status		
** **Retired (Reference category)	1.00	
** **Unemployed	1.02 (0.65–1.62)	.926
** **Working in healthcare sector	0.27 (0.17–0.43)	<.001**
** **Student	0.76 (0.49–1.20)	.236
** **Working outside healthcare sector	0.81 (0.51–1.29)	.380
Current smoker		
** **No (Reference category)	1.00	
** **Yes	0.94 (0.78–1.12)	.499
Have comorbidities history		
** **No (Reference category)	1.00	
** **Yes	1.05 (0.85–1.30)	.636

* *P* < .05.

** *P* < .001.

## 4. Discussion

Exploring the preparedness and knowledge of the public on emergency medicine requires understanding the extent to which the community is equipped with information and resources. The finding of the public knowledge on emergency services revealed that 81.4% of the participants can identify nearby emergency care service. Knowing the nearby emergency service is vital for individual’s safety, as Nicholl and his colleagues suggested that longer travel distance to emergency care is associated with higher mortality, with a 2% relative increase per kilometer and a 1% absolute increase per 10 km.^[34]^

On the other hand, only 48.1% was able to identify the rescue service with Saudi Arabia being the most aware country among participating countries (53.4%). Rescue services, which provide immediate care and safely extract individuals from critical situations, often start with an emergency medical call. Aljabri and Albinali, who explored public awareness of the emergency medical services phone number in Saudi Arabia, found a close result where 66% of participants reported awareness of the emergency medical services phone number.^[35]^ Inadequate knowledge in emergency care may indicate a limited EHL among participants, which in turn is associated with more frequent and improper health care usage, including emergency care.^[12,36]^

Limited health literacy in emergency care was also identified in the present study as reported by the participants regarding their capabilities of dealing with emergency cases. It was found that the highest degree of difficulty reported by the study participants was the difficulties they face to evaluate when to use appropriately utilize emergency medical services (39.6%). This result could be attributed to many factors such as the lack of knowledge about symptoms or medical conditions that warrant emergency care or limited access to reliable health information. Some patients, for example, use the internet, instead of medical consultation, to seek information regarding urgent healthcare conditions,^[37]^ while others rely on family or friends for medical guidance.^[38]^ Another factor could be the over-reliance on emergency services for less urgent indications.^[39]^

However, participants in the present study demonstrated a relative awareness of whom to contact in the event of a medical emergency (difficulties are found only in 29.3%), likely because they typically contact specialists based on specific symptoms. For instance, individuals experiencing eye conditions would seek ophthalmologist, while those with respiratory issues would turn to pulmonology care, depending on the severity.

The present study showed that majority of the study participants (75.1%) demonstrated problematic EHL, a result that significantly differs from findings in other countries. For example, in France, only 38% of participants exhibited problematic EHL,^[40]^ in Switzerland, the percentage was 23.5%,^[41]^ while in Spain, the figure was as low as 5.1%.^[42]^ This discrepancy suggests potential cultural, educational, and healthcare system differences that may influence EHL levels across Western and Middle Eastern countries. Specifically, among the participating countries, the lowest mean public EHL score (2.69 (SD: 0.50)) was observed for Kuwait, while Bahrain had the highest (2.89 (SD: 0.56)). Bahrain higher EHL might be accredited to relatively low population number which facilitate targeted and effective national health awareness programs.^[43]^

Sufficient EHL was more pronounced among males which is inconsistent with several studies in the Western literature that found a better EHL level in females.^[15,44,45]^ Females in Middle Eastern Countries may still face cultural and religious barriers that influence the use of healthcare services,^[46]^ indirectly limiting their exposure to health information.

Expectedly, public emergency literacy was higher among those who work in the health sector, likely due to their professional training and experiences, and regular access to health-related information.

Sufficient EHL was also demonstrated for young participants aged 24 to 30 years and elderly participants aged 61 years and older. These 2 age groups most frequently engage with health information due to life transitions or internet use (in case of those aged 24–30 years)^[40]^ and having more direct experience with the emergency care due to chronic conditions or age-related health concerns (in case of elderly).^[47]^

In the present study, education level and the socioeconomic status were found to be associated with EHL. These results are consistent with findings in the literature.^[44,48]^ Individuals with low educational level may find difficulties in understanding medical concepts and health information, and those with low-income levels may have limited access to emergency or healthcare services.

It worth mentioning that differences in healthcare infrastructure, models of funding, and country policies can have a large influence on the outcomes of public emergency health literacy across the study participants from 6 Middle Eastern countries. In Saudi Arabia, the Ministry of Health has established national health insurance schemes and e-health services like the 937 Call Center and the “Mawid” app to facilitate higher public engagement with health information.^[49]^ Similarly, platforms like Altibbi, launched in Jordan, aim to serve Arabic-speaking users by providing credible medical information, which could contribute to higher health literacy in the region.^[50]^ Without considering such contextual variables, the potential to determine whether observed health literacy inequalities at the witnessed level are due to education deficiency or maybe systemic healthcare disparity is reduced.

Therefore, a more highly specific country-level analysis is warranted to elucidate how availability of healthcare, financing mechanisms, and government policy individually affect each nation’s public emergency health literacy. Future studies should address these points and examine the influence of healthcare infrastructure, models of funding, and country policies on public emergency health literacy. Although the present study permits a wider understanding of EHL across diverse populations within the 6 countries, it may not fully account for cultural, socioeconomic, and healthcare system differences between each country, thus may limit the generalizability of the findings across them. Besides, the limitations that are inherent in cross-sectional studies; such as the difficulties in determining the cause-and-effect relationships and the generalizability.

## 5. Conclusions

The findings of the present study indicate limited emergency public EHL among 6 of the Middle Eastern countries, highlighting the need for interventions to promote public knowledge and capabilities. In this context, action plans in countries where the gaps are most pronounced should be developed to promote EHL. These plans should target females, those with low education and socioeconomic status, those in age range of 30 to 60 years, and those who do not work in health sector.

## Acknowledgments

The authors would like to thank Isra University for supporting this research (Grant number 2 -20/2023/2024).

## Author contributions

**Conceptualization:** Esra’ O. Taybeh, Abdallah Y. Naser.

**Data curation:** Esra’ O. Taybeh, Abdallah Y. Naser.

**Formal analysis:** Abdallah Y. Naser.

**Funding acquisition:** Abdallah Y. Naser.

**Investigation:** Esra’ O. Taybeh, Abdallah Y. Naser, Adnan Taybeh, Zahra K Alsairafi, Hassan Alwafi, Sami Qadus, Rania Itani, Alaa A. Alsharif, Ahmed M Al Rajeh, Jaber S. Alqahtani, Abdulelah M. Aldhahir, Anan S. Jarab, Saeed Alqahtani, Abdolelah Jaradat, Louai Saloumi, Yosra J Alhartani, Asaleh El-Qasem, Amer Hamad Issa Abukhalaf, Salman Alzayani, Roua Awni Attallah Aldala’een, Ahmad H Aburizeq, Ahmad Khaleel Hijazi, Jamal Alyoussef Alkrad, Mohamed Bahlol.

**Methodology:** Esra’ O. Taybeh, Abdallah Y. Naser.

**Project administration:** Esra’ O. Taybeh, Abdallah Y. Naser.

**Resources:** Esra’ O. Taybeh, Abdallah Y. Naser, Zahra K Alsairafi, Hassan Alwafi, Sami Qadus, Rania Itani, Alaa A. Alsharif, Ahmed M Al Rajeh, Jaber S. Alqahtani, Abdulelah M. Aldhahir, Anan S. Jarab, Saeed Alqahtani, Abdolelah Jaradat, Louai Saloumi, Yosra J Alhartani, Asaleh El-Qasem, Amer Hamad Issa Abukhalaf, Salman Alzayani, Roua Awni Attallah Aldala’een, Ahmad H Aburizeq, Ahmad Khaleel Hijazi, Jamal Alyoussef Alkrad, Mohamed Bahlol.

**Software:** Abdallah Y. Naser.

**Supervision:** Esra’ O. Taybeh, Abdallah Y. Naser.

**Validation:** Esra’ O. Taybeh, Abdallah Y. Naser.

**Visualization:** Esra’ O. Taybeh, Abdallah Y. Naser.

**Writing – original draft:** Esra’ O. Taybeh, Abdallah Y. Naser, Adnan Taybeh, Zahra K Alsairafi, Hassan Alwafi, Sami Qadus, Rania Itani, Alaa A. Alsharif, Ahmed M Al Rajeh, Jaber S. Alqahtani, Abdulelah M. Aldhahir, Anan S. Jarab, Saeed Alqahtani, Abdolelah Jaradat, Louai Saloumi, Yosra J Alhartani, Asaleh El-Qasem, Amer Hamad Issa Abukhalaf, Salman Alzayani, Roua Awni Attallah Aldala’een, Ahmad H Aburizeq, Ahmad Khaleel Hijazi, Jamal Alyoussef Alkrad, Mohamed Bahlol.

**Writing – review & editing:** Esra’ O. Taybeh, Abdallah Y. Naser, Adnan Taybeh, Zahra K Alsairafi, Hassan Alwafi, Sami Qadus, Rania Itani, Alaa A. Alsharif, Ahmed M Al Rajeh, Jaber S. Alqahtani, Abdulelah M. Aldhahir, Anan S. Jarab, Saeed Alqahtani, Abdolelah Jaradat, Louai Saloumi, Yosra J Alhartani, Asaleh El-Qasem, Amer Hamad Issa Abukhalaf, Salman Alzayani, Roua Awni Attallah Aldala’een, Ahmad H Aburizeq, Ahmad Khaleel Hijazi, Jamal Alyoussef Alkrad, Mohamed Bahlol.
